# Temperature, pressure, and humidity SAW sensor based on coplanar integrated LGS

**DOI:** 10.1038/s41378-023-00586-0

**Published:** 2023-09-11

**Authors:** Xiaorui Liang, Lei Zhang, Qiulin Tan, Wenhua Cheng, Dan Hu, Shuang Li, Lin Jing, Jijun Xiong

**Affiliations:** 1 State Key Laboratory of Dynamic Measurement Technology, North University of China, Taiyuan, 030051 China; 2 Key Laboratory of Micro/nano Devices and Systems, Ministry of Education, North University of China, Tai Yuan, 030051 China; 3 School of Materials Science and Engineering, Nanyang Technological University, 50 Nanyang Avenue, Singapore, 639798 Singapore

**Keywords:** Sensors, Electrical and electronic engineering

## Abstract

This paper presents a surface acoustic wave (SAW) sensor based on coplanar integrated Langasite (LGS) that is fabricated using wet etching, high-temperature bonding, and ion beam etching (IBE) processes. The miniaturized multiparameter temperature‒pressure-humidity (TPH) sensor used the MXene@MoS_2_@Go (MMG) composite to widen the humidity detection range and improve the humidity sensitivity, including a fast response time (3.18 s) and recovery time (0.94 s). The TPH sensor was shown to operate steadily between 25–700 °C, 0–700 kPa, and 10–98% RH. Coupling issues among multiple parameters in complex environments were addressed by decoupling the Δf-temperature coupling factor to improve the accuracy. Therefore, this work can be applied to simultaneous measurements of several environmental parameters in challenging conditions.

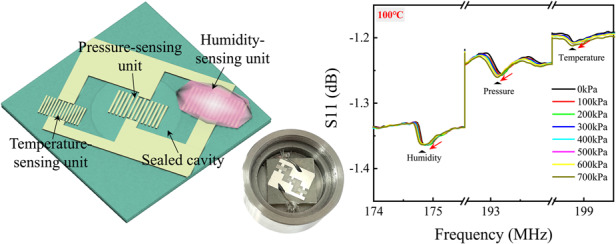

## Introduction

Complicated operational environments are often encountered in various machinery systems, such as those used in forest fires^1^, mining metallurgy, mine environmental monitoring^2^, industrial pipelines^3^, and aircraft^4^. Making matters more difficult, most state-of-art sensors are designed for measuring a single parameter^5–7^, whereas multiparameter sensors with small volume, high performance, and single-chip integration are more promising solutions in these conditions. For example, sensors for measuring temperature and pressure^8^, temperature and humidity^9,10^, temperature and strain^11,12^, temperature, pressure and humidity^13,14^, temperature, humidity and magnetic^15^ have been demonstrated. Specifically, Niladri Banerjee et al.^16^ demonstrated multiple sensors on a single substrate, including capacitive inertial sensors, capacitive absolute pressure sensors, resistive temperature sensors, and capacitive microphones. Jin Wang et al. ^17^ reported a multiparameter measurement sensor based on optical fiber for the simultaneous real-time detection of temperature, stress, refractive index, and humidity. Tan et al. ^13^ developed a wireless passive temperature, pressure, and humidity sensor incorporating a low-temperature co-fired ceramic (LTCC) to operate stably between 25-200 °C, 70-220 KPa, and 24-90% RH. Kou et al. ^14^ designed an integrated temperature-pressure-humidity sensor with a complementary split-ring resonator (CSRR) to operate at 25–300 °C, 10–300 kPa, and 20–90% RH. Lei Dong et al.^18^ presented a novel inductive structure coupled capacitive temperature, pressure, and relative humidity sensors with laminated inductors for stable operation under 15%-90% RH, 20-100 °C, and 50-110 kPa. However, these TPH sensors based on LC mutual inductance suffer from problems such as low integration and large size. In contrast, surface acoustic wave (SAW) sensors are presented in this work because of their straightforward construction, small size, good stability, and feasibility for multiparameter integrations. In addition, the two-dimensional nanomaterial graphite oxide (Go) film has oxygen-containing functional groups, including epoxy, hydroxyl, carboxyl, ester, and other active groups^19,20^. Specifically, molybdenum disulfide (MoS_2_) has vertically stacked nanostructures with Mo-S atoms for low interlaminar shear strength (mainly related to weak van der Waals forces)^21^, good heat resistance under high load and vacuum conditions, slow recovery/response time, and low sensitivity^22^. However, its surface lacks functional groups, making functionalization difficult^23^. MXene is an emerging two-dimensional conductive material with excellent conductivity and functional groups that can stably connect to fiber substrates^24^. However, its poor stability in aqueous media results in poor water adsorption^25^. MoS_2_ can compensate for voids and defects that form in the MXene stacking process^23^, while Go and MXene are two-dimensional materials with large surface areas, so the total effective area does not decrease when forming composite films^26^. Growing MoS_2_ film on the surface of Go film can effectively prevent the accumulation and aggregation of MoS_2_^27^. Prior analysis has shown that nanocomposites can effectively avoid defects in a single material, and combining the advantages of Go, MoS_2_, and MXene into a composite structure can greatly improve the hydrophilicity of the membrane. Thus, we chose the MXene@MoS_2_@Go (MMG) composite material as our humidity-sensitive material.

Langasite (LGS) piezoelectric crystal is a piezoelectric material. It exhibits high-temperature stability without pyroelectric or ferroelectric properties^28,29^, making it a piezoelectric material with high-temperature stability. Therefore, we selected LGS as the piezoelectric substrate for the SAW sensors. A TPH SAW sensor was designed with the pressure-sensing unit based on a sealed cavity structure. The multilayer MMG composite was characterized by scanning electron microscopy (SEM), transmission electron microscopy (TEM), Raman spectroscopy, and X-ray photoelectron spectroscopy (XPS). Experimental results show that the TPH sensor can operate steadily in the environment of 25–700 °C, 0–700 kPa, and 11–98% RH. Moreover, a multiparameter decoupling algorithm was developed to address multiparameter coupling issues, and the maximum error between the measured and demodulated value values was 5.5%. The resulting TPH SAW sensor could detect a wide range of applications for multiparameter, simultaneous monitoring of complex environments.

## Integrated TPH sensor structure

**Fig. 1:**
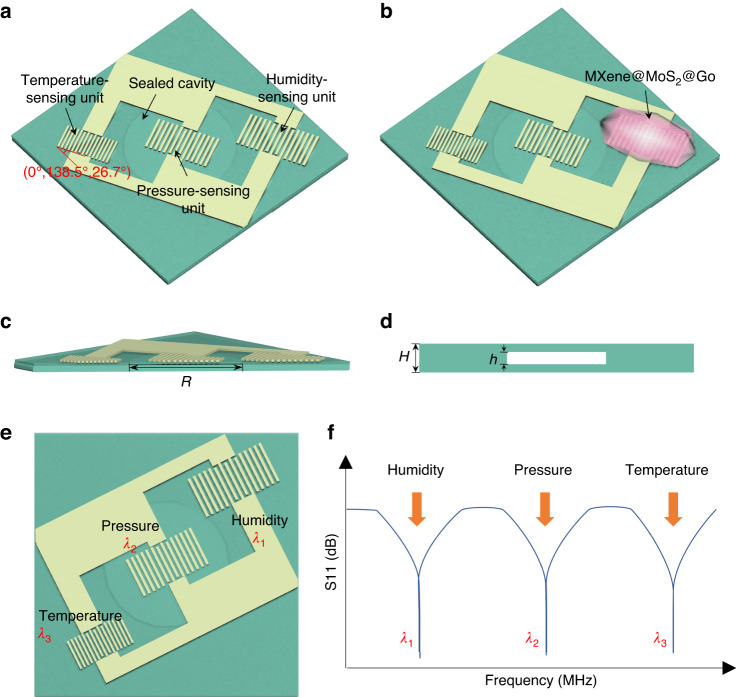
Structural design analysis of SAW sensors. **a**–**d** Schematic diagram of the TPH structure. **e** TPH structures with different wavelengths. **f** The S_11_ vs. frequency plot shows the separation of frequencies

The TPH sensor has a coplanar structure with temperature, pressure, and humidity sensing units, as shown in Fig. 1a–d. The substrates are LGS wafers of size 20 mm × 20 mm × 0.5 mm and 20 mm × 20 mm × 0.3 mm with cut angles of (0°, 138.5°, 0°). The pressure-sensing unit is located on the top surface of the sealed cavity, where the diameter and height of the cavity are 10 mm and 0.1 mm, respectively. The MMG composite material is applied on a humidity-sensing unit as the sensitive material.

The LGS crystal exhibits anisotropic properties, with variations in physical, chemical, and other characteristics along different directions. The optimal cut and propagation direction for different sensing units must be carefully designed. A previous study has shown that optimal sensitivity can be achieved for temperature and pressure sensors for crystals with (0°, 138.5°, 25°) and (0°, 138.5°, 30°) cuts, respectively^30^. Due to the symmetry of the LGS, a reasonable layout design can be achieved with multiple parameters, as shown in Fig. S1. However, an integrated sensor is simultaneously affected by temperature, pressure, and humidity, where temperature often exerts a greater influence on sensor sensitivity on external factors than the other two parameters. Considering the cross-coupling effect of temperature and other variables, our three sensing units included the optimal temperature sensitive cut of (0°, 138.5°, 26.7°) so that all sensing units are similarly impacted by temperature to ensure good consistency under temperature, pressure, and humidity variations.

The problem of crosstalk between the sensing frequencies of each sensing unit is addressed using a design method that does not intersect in the sensing frequency range of individual sensor units. As shown in Eq. (1), the frequency of the SAW is determined by its wavelength and wave speed. Therefore, it is possible to separate the frequency of sensing units by adjusting the wavelength. In the prototype design, the wavelengths of the temperature-sensing unit, pressure-sensing unit, and humidity-sensing unit, ${\lambda }_{3}$, ${\lambda }_{2}$, and ${\lambda }_{1}$, are 13.6 μm, 14 μm, and 15.6 μm, respectively. The sensing structures with different wavelengths are shown in Fig. 1e, and the separation of frequencies is shown in the S_11_ vs. frequency plot in Fig. 1f.

1 $$f=\frac{v}{\lambda }$$

## Material and methods

### Fabrication of MMG Heterostructure Membranes

Ti_3_C_2_T_x_ was converted into Mxene solution (2 mg/ml, Vertene technology) by selectively etching Al atoms with Ti_3_AlC_2_. The Hummers method was employed to produce the Go solution (2 mg/ml, layer: 1-6, diameter < 4 μm, Xianfeng nanotechnology). The MoS_2_ solution was obtained by adding MoS_2_ powder (Xianfeng nanotechnology) to deionized water (50 ml). Then, the solution was ultrasonically dispersed at 30 °C for 2 h to weaken the interaction between MoS_2_ nanoparticles and continuously stirred with a magnetic stirrer at 25 °C and 1000 r/min for 2 h to obtain a dispersion of 2 mg/ml MoS_2_.

**Fig. 2:**
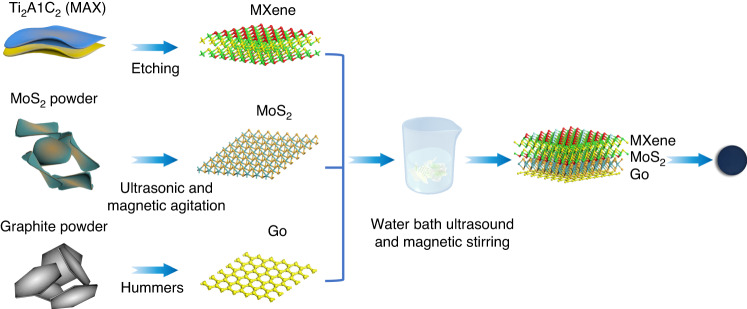
The fabrication process of the MMG hybrid

By using a micropipette, 2 ml of MXene dispersion, 6 ml of Go dispersion, and 2 ml of MoS_2_ dispersion were poured into a beaker and mixed. Then, 10 ml of deionized water was additionally poured into the beaker. For mixing, the beaker was placed in an ultrasound water bath and sonicated at 25 °C for 24 h to obtain a uniform MMG dispersion. Next, the evenly mixed MMG dispersion solution was put into the vacuum suction filtration device for filtration, fully cleaned, and filtered with deionized water. All excess water and foam were efficiently pumped and filtered. Subsequently, the filter membrane was placed on a heating table at 80 °C for drying and placed into ethanol solution. The MMG membrane was peeled from the filter membrane with tweezers. A 2 cm × 2 cm shrink film was taken and treated by plasma for 10 minutes, followed by transferring the MMG film to the shrink film. After natural drying, it was placed in a vacuum drying oven for drying at 135 °C for 15 min. Finally, the shrink film was removed and put into a dichloromethane solution to obtain the MMG film by a peeling process. The detailed process of the MMG hybrid is shown in Fig. 2.

### Fabrication of the integrated TPH sensor

**Fig. 3:**
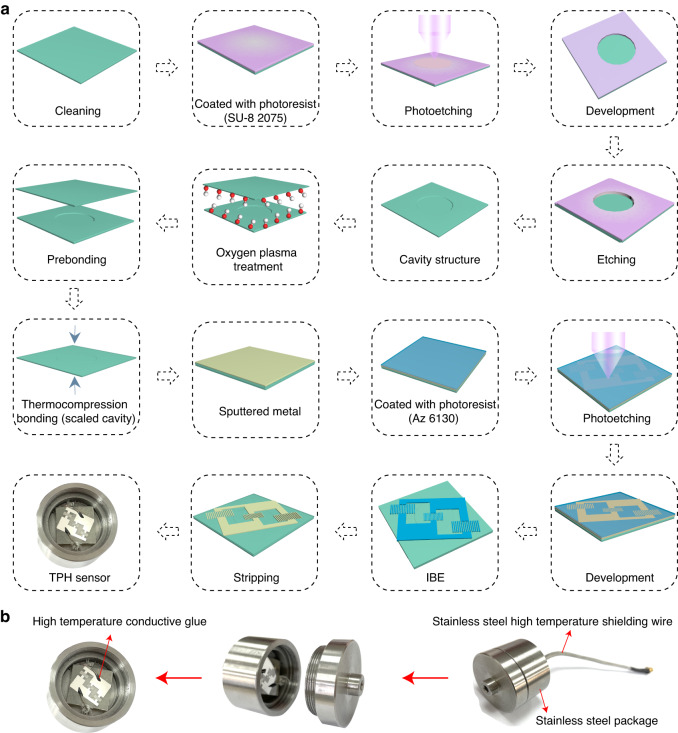
Fabrication of the integrated TPH sensor. **a** The fabrication process of the integrated TPH sensor. **b** Packaged structure of integrated TPH sensor

Figure 3a shows the detailed fabrication process of the LGS-based and integrated TPH sensor. First, a cavity with a diameter of 10 mm and a height of 0.1 mm was formed by a wet etching process, with a 1:1 mixture solution of HCl and H_3_PO_4_ serving as the etchant. To prebond the two substrates, the substrate with a cavity etched on its surface and the other substrate’s polishing surface was treated with oxygen plasma. Subsequently, a sealed cavity was produced by high-temperature direct bonding. The surface of the substrate with a sealed cavity achieved surface patterning by an ion beam etching (IBE) process. The IBE process includes (1) sputtering the metal layer, (2) coating photoresist, (3) photolithography, (4) developing, (5) IBE, and (6) stripping. The metal electrode material of the sensor was platinum (Pt) with a thickness of 200 nm. Finally, the prepared sensor was packaged, and the final packaged structure is shown in Fig. 3b.

### Characterization

The surface characteristics of different two-dimensional nanomaterials were investigated by SEM and TEM. The heterogeneous hierarchical structures of multilayer composite films were analyzed, and the formation of multilayer composite thin films was demonstrated by Raman spectra analyses. The elemental composition and surface state of the films were characterized by XPS to analyze the hydrophilicity of the multilayer composite films. The excitation source was an Al ka ray (hv=1253.6 eV) with an electron emission angle of 45°.

## Experimental results and discussion

### MMG nanomaterial analysis

**Fig. 4:**
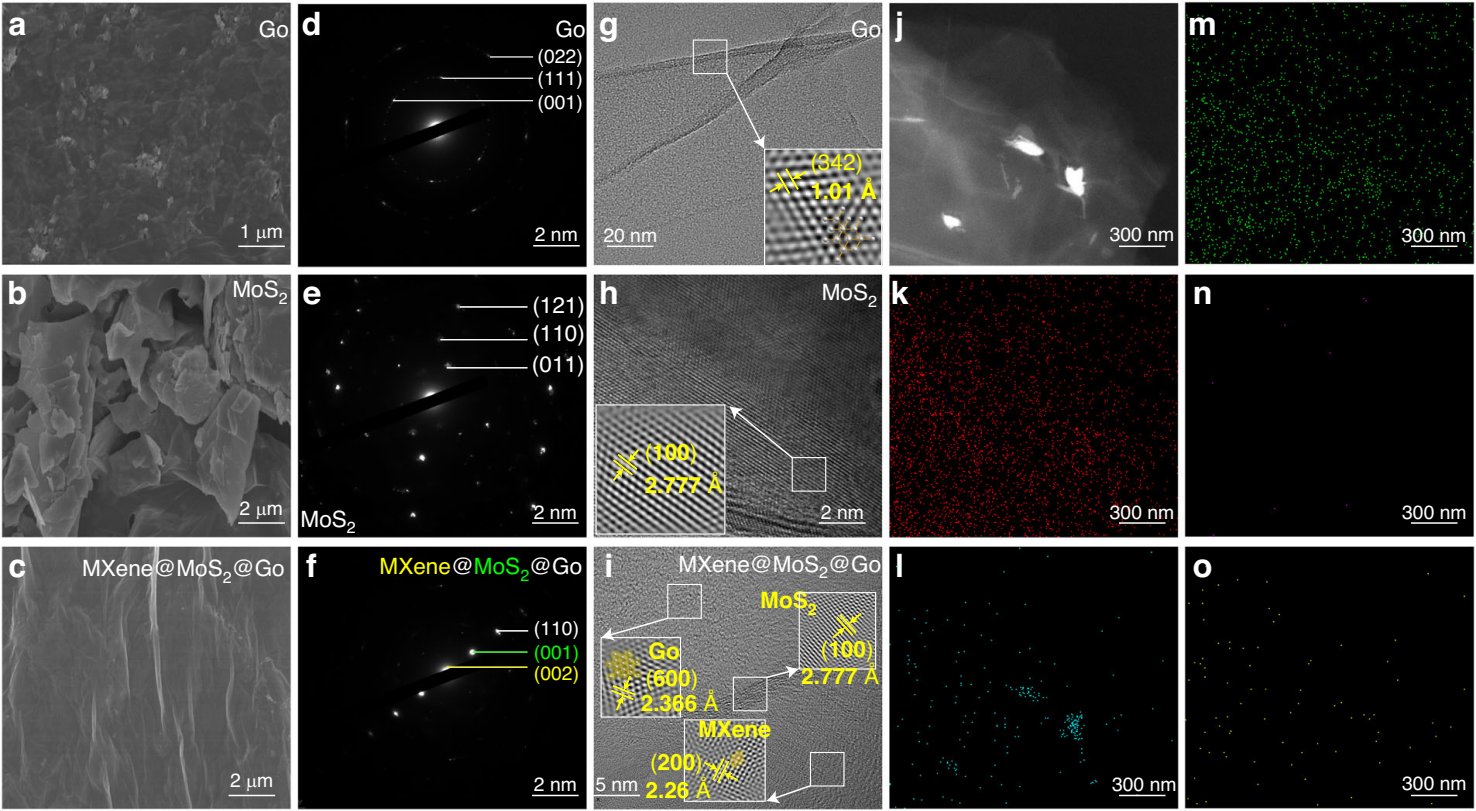
Microscopic characterization of two-dimensional nanomaterials. Go, MoS_2_, MXen@MoS_2_@Go two-dimensional nanomaterials **a**–**c** SEM characterization diagram **d**–**f** SAED diffraction pattern **g**–**i** High-resolution TEM images. **j**–**o** The map of elements

The two-dimensional MMG nanocomposites were analyzed. The SEM surface characterization results of the pure Go film, pure MoS_2_ film, and MMG composite film are shown in Fig. 4a–c. The surface of the pure Go film exhibited a certain number of folds, as shown in Fig. 4a, which is a typical surface structure of the Go film. Figure 4b presents the SEM image of pure MoS_2_, consisting of large and dispersed particles with a microscale diameter. As illustrated in Fig. 4c, the surface of the MMG membrane exhibits wrinkles, which are the inherent feature of two-dimensional nanomaterials. This feature reflects that the prepared MXene, MoS_2_, and Go films have homogenous and stable structures. The regional electron diffraction (SAED) diagram of pure Go, pure MoS_2,_ and MMG two-dimensional heterostructures is shown in Fig. 4d–f. The three highest-intensity diffraction rings in Fig. 4f correspond to the (002) crystal plane of MXene, the (001) crystal plane of MoS_2_, and the (110) crystal plane of Go. Figure 4g demonstrates that the lattice spacing of the pure Go (342) crystal plane is 1.01 Å. The lattice spacing of the pure MoS_2_ (100) crystal plane in Fig. 4h is 2.777 Å. In addition, the lattice fringes of 2.366 Å, 2.777 Å, and 2.26 Å in Fig. 4i are attributed to the Go (600) crystal plane, MoS_2_ (100) crystal plane, and Mxene (200) crystal plane, respectively. Figure 4i shows that Go, MoS_2_, and MXene form a multilayer structure, which is consistent with the findings of the SAED diffraction pattern. The movement of charge carriers is facilitated by the additional contact surfaces of this multilayer structure^31,32^.

The TEM mapping of the MMG composite is shown in Fig. 4j–o. The elements C, Ti, O, Mo, and S are evenly distributed, indicating that MXene, MoS_2_, and Go are homogeneously mixed. These findings demonstrate that the MMG composites generate layered and heterogeneous structures.

**Fig. 5:**
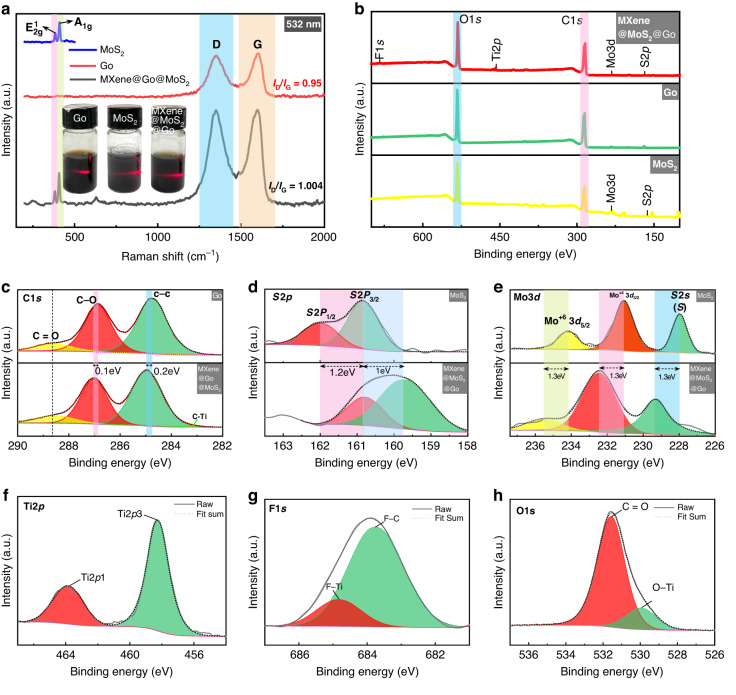
Different analysis methods for MMG composite film. **a** The Raman spectrum. **b-h** XPS analysis of the MMG composite surface

The Raman spectra of the Go film, MoS_2_ film, and MMG composite film samples are illustrated in Fig. 5a (laser wavelength: 532 nm). All samples were recorded with wavelengths between 200 and 2000 cm^-1^. There are two peaks at 360 cm^-1^ and 430 cm^-1^ in the Raman spectrum of the MoS_2_ thin film, corresponding to the in-plane${E}_{2g}^{1}$ and out-of-plane ${A}_{1g}$ vibrations, respectively^33^. These two peaks are related to the vibration of the 2H-MoS_2_ phase^34^. The Raman spectrum of the Go film has a D-peak at 1347 cm^−1^, which is due to the symmetric stretching vibration of carbon atom Sp^2^ hybridization, indicating the presence of vacancies, edge unsaturation, and structural defects in Go films. At the defect edge, there are many oxygen-containing functional groups that can adsorb water molecules from the surrounding air^35^. The G-peak (1600 cm^−1^) denotes the sp^2^ C-C bond, which is caused by the in-plane stretching vibration of the sp^2^ hybridization in the carbon atoms^36^. In addition, the MMG composite film contains the ${E}_{2g}^{1}$ and ${A}_{1g}$ band characteristic peaks of MoS_2_ and the D and G band characteristic peaks of the Go film. The coexisting peaks in the Raman spectra indicate that the MMG composite film is successfully prepared. Additionally, the intensity ratio (I_D_/I_G_) between the D and G peaks is 1.004, which is slightly higher than that of the Go film (0.95), indicating that there are many defects in the nanocomposite film, which serve as active adsorption sites for water molecules^27,37^.

As shown in Fig. 5b, the elemental composition and surface states of Go, MoS_2,_ and MMG films were characterized by XPS. MMG is represented mainly as six elements, namely, C, O, Mo, S, Ti, and F. After HF etching, the Al disappears, so the surface exhibits good hydrophilicity^38^. Additionally, we confirmed that these elements coexist in MMG films to form heterogeneous structures of hybridization^39^. Figure 5c shows the Mo3d high-resolution XPS spectra of MoS_2_ and MMG films. The Mo3d spectra of MoS_2_ peaking at 234.2 eV and 231.1 eV correspond to Mo^+6^ 3d5/2 and Mo^+4^ 3d5/2, respectively. Compared with the MoS_2_ film, the binding energy of the MMG composite film increases by 1.3 eV. The composite film has stronger electronegativity than pure MoS_2_, demonstrating that the MoS_2_ nanosheet was successfully inserted into the MXene interlayer space^40^. Additionally, the peak at 229.3 eV is S2s in MoS_2_. The high-resolution XPS spectra of S2p are presented in Fig. 5d. The MMG spectrum has two characteristic peaks at 160.8 eV and 159.7 eV, corresponding to S2p1/2 and S2p3/2, respectively^34^. The composite film exhibits a clear shift for pure MoS_2_, which shows a strong interaction of MoS_2_/MXene^41^. The spectrum of C1s is presented in Fig. 5e. The characteristic peaks of C-O and C-C are MMG at 287 eV and 285 eV, respectively. Compared with the Go film, the MMG film’s binding energy is increased; also, the surface has richer oxygen-containing functional groups, which contribute to improved hydrophilicity^42^. At a binding energy of 291 eV, a new characteristic peak (C-Ti) appears, and a C-Ti bond is created by Mxene and Go, which is consistent with the results from the Raman spectrum (Fig. 5a)^43^. In Fig. 5f, the Ti2p spectrum is divided into two peaks, where 463.9 eV and 458.3 eV are the characteristic peaks of Ti2p1 and Ti2p3, respectively^44^. Figure 5g shows the high-resolution XPS map of F1s with the characteristic F-Ti and F-C peaks at 463.9 eV and 458.3 eV, respectively. The high-resolution XPS spectra of O1s are displayed in Fig. 5h. The O1s high-resolution spectrum consists of two peaks^45^, with O-Ti at 529.9 eV and C = O at 531.6 eV^46^.

### The test and analysis of the humidity sensor

**Fig. 6:**
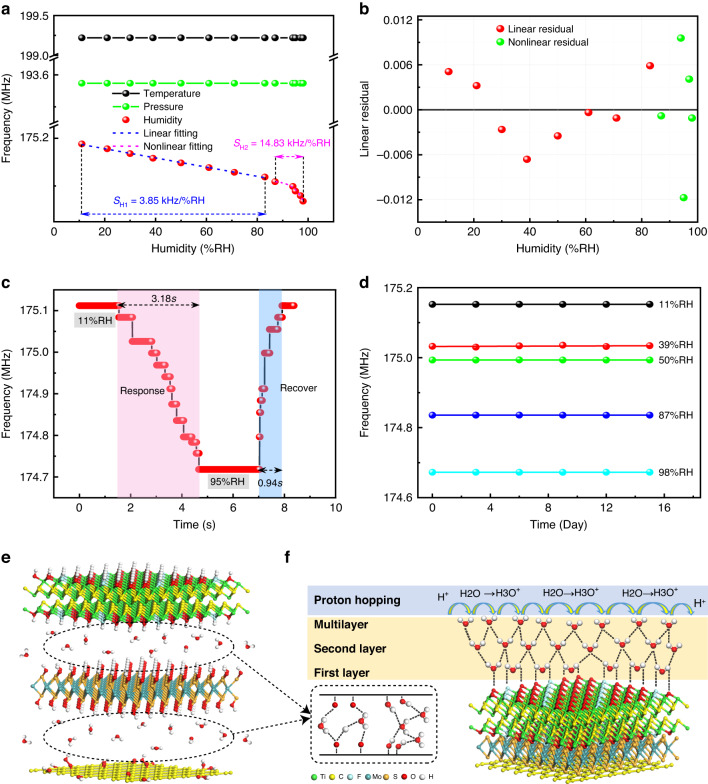
Test results and mechanism analysis of humidity sensors. **a** The relationship between humidity and frequency for each sensing unit under ambient temperature and pressure. **b** The measurement error of the humidity-sensing unit. **c** The response and recovery time of the humidity-sensing unit. **d** The stability of the humidity-sensing unit based on the MMG nanocomposite-sensitive film. **e**, **f** The sensitivity mechanism of the humidity-sensing unit

The relationship between humidity and the frequency of temperature, pressure, and humidity-sensitive units at 11%-98% RH under 25 °C and in an ordinary pressure environment is illustrated in Fig. 6a. The results demonstrate that the resonant frequency of humidity-sensitive units decreases with increasing humidity, while the resonance frequencies of the temperature- and pressure-sensitive units do not shift.

The sensitivity ${S}_{H}$ of the humidity sensor is calculated as:

2 $${S}_{H}=\frac{\varDelta {f}_{{P}_{0},H}^{{T}_{i}}}{\varDelta H}$$

where $\varDelta {f}_{{P}_{0},H}^{{T}_{i}}$ is the frequency change of the humidity sensing unit under different humidity conditions at temperature ${T}_{i}$ and pressure ${P}_{0}$ and $\varDelta H$ is the change in humidity under temperature ${T}_{i}$ and pressure ${P}_{0}$.

According to Formula (2), the sensitivity of the humidity-sensitive unit is 3.85 kHz/%RH at low humidity (11%–83% RH) and 14.83 kHz/%RH at high humidity (87%–98% RH). As demonstrated in Fig. 6a, the humidity-frequency curve of the humidity-sensing unit was fit to various trends, and the linear fitting residual (for low humidity) and nonlinear fitting residual (for high humidity) were obtained, as shown in Fig. 6b. The maximum variations for linear and nonlinear fitting were 0.59% and 1.17%, respectively. Figure 6c shows the response and recovery time of the humidity-sensitive unit at 25 °C and 11% RH-95% RH. The response (adsorption) and recovery (desorption) time periods were 3.18 s and 0.94 s, respectively. Meanwhile, the response and recovery behaviors of the humidity sensor were tested in two saturated salt solutions (LiCl: 11.3% and KNO_3_: 95%). At a temperature of 25 °C, our sensor was first placed on top of the LiCl-saturated solution. After stabilization, it was quickly transferred to the top of the KNO_3_-saturated solution to evaluate the response time. After the sensor was stabilized in the KNO_3_-saturated solution, it was quickly transferred to the top of the LiCl solution to evaluate the recovery time. In this experiment, it is known that the proton hydroxyl desorption process is faster than the adsorption process. The possible reasons for the desorption time being shorter than the adsorption time include the following: (i) The contact between water molecules and MXene depends on weak physical adsorption, such that water molecules during the relaxation process could easily leave the surface of MMG. (ii) MoS_2_ mainly absorbs water molecules through physical adsorption on the surface with weak bonding forces such that it can recover quickly in a short time when the water content decreases. MoS_2_ is in the MXene and Go sandwich to increase its surface volume and provide more marginal active sites, which is conducive to the release of water molecules. (iii) Water molecules are not immersed in deeper membranes, and proton jumps occur less frequently. The developed sensor has superior performance to those of SAW humidity sensors based on other two-dimensional nanomaterials, as shown in Table 1. To evaluate the sensor’s dependability, the humidity-sensitive unit based on the MMG nanocomposite-sensitive film was tested continuously for half a month, and the result was recorded every three days, as shown in Fig. 6d. The data show that the sensor’s resonance frequency does not vary significantly under different humidity conditions of 11% RH, 39% RH, 50% RH, 87% RH, and 98% RH, indicating good stability of the sensor.

**Table 1:** The SAW humidity sensing characteristics based on different two-dimensional nanomaterials

Materials	RH Meas. range	Res. Time	Rec. time	Ref.
BC	30%-93%	12 s	5 s	^54^
Go	15%-80%RH	9 s	9 s	^19^
SiO_2_	10%-70%RH	6 s	21.3 s	^55^
Go	20%-70%RH	9 s	12 s	^20^
ZnO/ZnO/Quartz	1%-80%RH	60 s	40 s	^56^
3DAG/PVA/SiO_2_	0%-90%RH	24 s	14.4 s	^57^
Go/MoS_2_	20%-95%RH	6.6 s	3.5 s	^58^
MMG	11%-95%RH	3.18 s	0.94 s	This work

### Sensitivity of the humidity-sensitive unit

The MMG nanocomposite is used for the humidity-sensitive layer to leverage the advantages of each constituent material. The MXene surface is hydrophilic because it is rich in functional groups, including hydroxyl (-OH), oxygen (=O), fluorine (-F), and other terminal groups^47,48^. MoS_2_ has great adsorption capacity and a high surface-to-volume ratio, and it can be employed as a supporting layer, improving the diffusion of water molecules in sensitive films^49,50^. Go contains a large number of oxygen-containing groups, such as epoxy, hydroxyl, carboxyl, ester groups, and other active groups and defective sites, which act as absorption sites for water molecules^51^. Therefore, the absorbability of nanocomposite materials is strong, and water molecules load as the humidity increases, leading to a frequency shift in the response.

The sensing mechanism of the humidity-sensing unit is shown in Fig. 6e, f. In low-humidity environments, the surface of the nanocomposite material is rich in hydrophilic functional groups to adsorb water molecules by double hydrogen bonding. The water molecules are immobilized as the first layer of physical adsorption. As the humidity increases, the second layer of water molecules is absorbed by the first layer of water by a single hydrogen bond as the second layer of physical adsorption^52^. When an increasing number of water molecules are adsorbed (multilayer physical adsorption) and gradually reach a saturation state, a water film layer forms on the surface of the humidity-sensing material. When an electric field is present, water molecules are protonated (H_2_O + H^+^ → H_3_O^+^) to generate hydronium ions^53^. Simultaneously, free water seeps into the interlayer of the hygro-sensitive material, increasing its mass and resulting in a significant frequency shift.

### Test and analysis of the integrated TPH sensor

Fig. S2a compares the three separate SAW sensors with the integrated sensor. The data show that the S11 values of the three separated devices are higher than those of the integrated sensor. Meanwhile, because the Q-factor of the integration sensor is small, the impedance values of each part of the integration sensor are enlarged. The impedance curve of the device is presented in Fig. S2b, and the distribution of resonance peaks can be observed, where f_r_ is the resonator and f_a_ is the anti-resonance. In addition, the Q-factors of the three individual sensors for humidity, pressure, and temperature are approximately 800, 1200, and 1900, respectively, corresponding to the Q-factors of the integrated sensor being approximately 550, 1000, and 900, indicating that the performance of the integrated devices is inferior to those of the three separated SAW devices. However, the integrated sensor has the advantage of a small form factor.

**Fig. 7:**
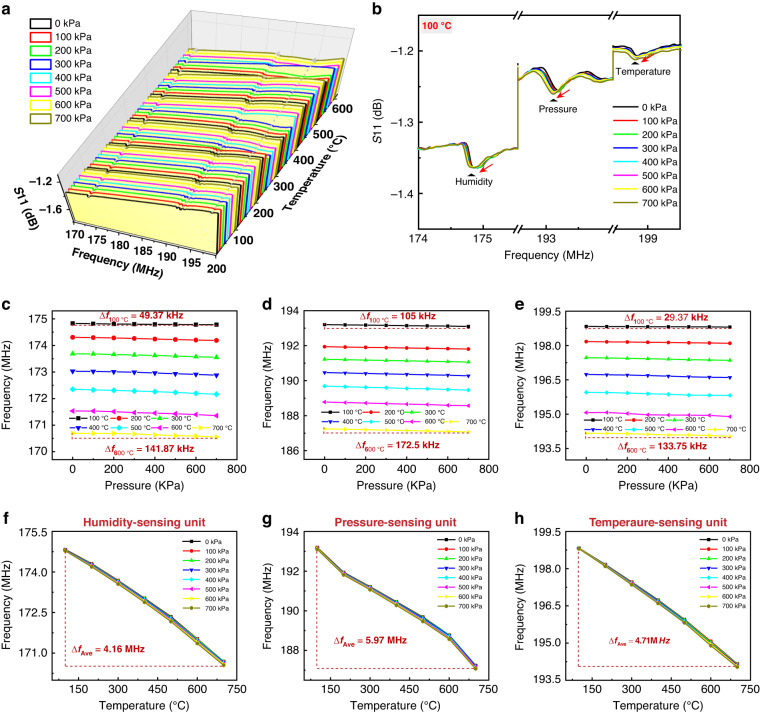
Test results of the integrated TPH sensor. **a** The 3D waterfall plot of frequency versus S11 for temperature and pressure in the conditions of 0–700 kPa and 100–600 °C under the same humidity. **b** Plot of frequency versus S11 for TPH sensors in the condition of 0–700 kPa at 100 °C under different pressures. **c** The humidity-sensing unit; **d** the pressure-sensing unit; and **e** the temperature-sensing unit results for the frequency vs. pressure tests at different temperatures of 0–700 kPa. Frequency vs. temperature curves of the **f** humidity-sensing unit, **g** pressure-sensing unit, and **h** temperature-sensing unit under different pressures within 100–700 °C

The integrated TPH sensor was tested and examined in the conditions of 0-700 kPa and 100-700 °C under the same humidity environment. The relationship between the frequency and the S11 value of the temperature-, pressure-, and humidity-sensitive units is illustrated in a 3D waterfall diagram in Fig. 7a. The diagram shows that the curve has three clear resonant peaks corresponding to the resonant frequencies of the temperature, pressure, and humidity-sensing units. Meanwhile, Fig. 7a shows that the resonant peaks of the three sensing units change with temperature. This occurs because multiple sensing units are integrated on the same substrate, each of which is affected by temperature. To observe the influence of different pressures on the sensing units at the same temperature, the relationship between frequency and S11 values at 100 °C and different pressures in the 3D waterfall plot is enlarged, as shown in Fig. 7b. The T, P, and H sensing units are all affected by pressure, while pressure has the greatest influence on the pressure-sensing unit.

Furthermore, in the same humidity environment, the impact of temperature and pressure on the integrated device was investigated. The relationships between the frequency vs. pressure results for the humidity, pressure, and temperature sensing units under various temperatures are presented in Fig. 7c–e, respectively.

These results demonstrate that there is a linear relationship between pressure and frequency in all cases. The pressure-sensing unit is located on the surface of the cavity, where there is a greater variation in stress. Thus, the pressure has the greatest impact on the pressure-sensing unit, resulting in a maximum deviation of the resonant frequency of 172.5 kHz and a sensitivity of 287.5 Hz/kPa. The frequency deviations of the temperature and humidity sensing units caused by pressure are relatively small. Figure 7f–i shows the relationship between the temperature vs. frequency of the TPH-sensing units under different pressures. The results indicate that under the same pressure, there is a quadratic relationship between temperature and frequency. Similarly, the temperature and humidity-sensing units are affected by temperature variations. In general, an increase in temperature results in a decrease in frequency. This occurs because the temperature- and humidity-sensitive units are based on the same cut of the substrate. The pressure-sensitive unit is greatly affected by temperature because it is affected by both stress and temperature. Since multiple parameters are coupled to each other, it is necessary to reduce temperature effects.

### Temperature‒pressure-humidity decoupling algorithm

The sensitivity ${S}_{P}$ of the pressure-sensing unit at a certain temperature and humidity is:

3 $${S}_{P}=\frac{\Delta {f}_{P,{H}_{i}}^{{T}_{i}}}{\Delta P}$$

where $\Delta {f}_{P,{H}_{i}}^{{T}_{i}}$ is the frequency change of the pressure-sensing unit under different pressures at a temperature of ${T}_{i}$ and humidity of ${H}_{i}$; $\varDelta P$ is the pressure change under the same pressure of ${T}_{i}$ and humidity of ${H}_{i}$.

In the absence of pressure (0 KPa) and certain humidity, the sensing result is only influenced by temperature, and the change in frequency can be expressed as:

4 $$\frac{\Delta {f}_{{P}_{0},{H}_{0}}^{T}}{{f}_{{P}_{0},{H}_{0}}^{{T}_{0}}}=\frac{{f}_{{P}_{0},{H}_{0}}^{T}-{f}_{{P}_{0},{H}_{0}}^{{T}_{0}}}{{f}_{{P}_{0},{H}_{0}}^{{T}_{0}}}=TC{F}_{1}\Delta T+TC{F}_{2}\Delta {T}^{2}$$

where $\varDelta {f}_{{P}_{0},{H}_{0}}^{T}$ is the change in frequency at different temperatures when under a pressure of ${P}_{0}$ and humidity of ${H}_{0}$; ${f}_{{P}_{0},{H}_{0}}^{{T}_{0}}$ is the initial frequency under a temperature of ${T}_{0}$, a pressure of ${P}_{0}$, and a humidity of ${H}_{0}$. TCF_1_ and TCF_2_ are the first- and second-order frequency temperature coefficients, respectively. However, under the influence of temperature, pressure, and humidity, the relative frequency varies as:

5 $$\begin{array}{c}\frac{\varDelta {f}_{P,H}^{T}}{{f}_{{P}_{0},{H}_{0}}^{{T}_{0}}}=\frac{{f}_{P,H}^{T}-{f}_{{P}_{0},{H}_{0}}^{{T}_{0}}}{{f}_{{P}_{0},{H}_{0}}^{{T}_{0}}}=\frac{{f}_{P,H}^{T}-{f}_{{P}_{0},H}^{T}+{f}_{{P}_{0},H}^{T}-{f}_{{P}_{0},{H}_{0}}^{T}+{f}_{{P}_{0},{H}_{0}}^{T}-{f}_{{P}_{0},{H}_{0}}^{{T}_{0}}}{{f}_{{P}_{0},{H}_{0}}^{{T}_{0}}}\\ =\frac{\varDelta {f}_{P,H}^{T}+\varDelta {f}_{{P}_{0},H}^{T}+\varDelta {f}_{{P}_{0},{H}_{0}}^{T}}{{f}_{{P}_{0},{H}_{0}}^{{T}_{0}}}=\frac{{{\rm{S}}}_{{\rm{p}}}\times \varDelta P}{{f}_{{P}_{0},{H}_{0}}^{{T}_{0}}}+\frac{{{\rm{S}}}_{{\rm{H}}}\times \varDelta H}{{f}_{{P}_{0},{H}_{0}}^{{T}_{0}}}+TC{F}_{1}\varDelta T+TC{F}_{2}\varDelta {T}^{2}\end{array}$$

According to Formula (5), in a certain humid environment, under the influence of both temperature and pressure, the relative frequency changes as:

6 $$\frac{\varDelta {f}_{P,{H}_{i}}^{T}}{{f}_{{P}_{0},{H}_{0}}^{{T}_{0}}}=\frac{{{\rm{S}}}_{{\rm{p}}}\times \varDelta P}{{f}_{{P}_{0},{H}_{0}}^{{T}_{0}}}+TC{F}_{1}\varDelta T+TC{F}_{2}\varDelta {T}^{2}$$

According to Formula (5), under the pressure of ${P}_{0}$, the relative frequency changes under the influence of both temperature and humidity as:

7 $$\frac{\varDelta {f}_{{P}_{0},H}^{T}}{{f}_{{P}_{0},{H}_{0}}^{{T}_{0}}}=\frac{{{\rm{S}}}_{{\rm{H}}}\times \varDelta H}{{f}_{{P}_{0},{H}_{0}}^{{T}_{0}}}+TC{F}_{1}\varDelta T+TC{F}_{2}\varDelta {T}^{2}$$

Because the temperature, pressure, and humidity-sensing units are integrated on the same substrate, the first-order frequency temperature coefficient is the same as the second-order frequency temperature coefficient. According to Formulas (4), (5), and (7), the output frequency of the pressure-sensing unit is:

8 $${f}_{P}={f}_{{P}_{0},{H}_{0}}^{{T}_{0}}+\varDelta {f}_{P,H}^{T}-\varDelta {f}_{{P}_{0},H}^{T}+\varDelta {f}_{{P}_{0},{H}_{0}}^{T}={f}_{{P}_{0},{H}_{0}}^{{T}_{0}}+{S}_{P}\times \varDelta P+\varDelta {f}_{{P}_{0},{H}_{0}}^{T}$$

The pressure-sensing unit is subject to pressure changes as follows:

9 $$\varDelta P=\frac{|{f}_{P}-{f}_{{P}_{0},{H}_{0}}^{T}|}{{S}_{P}}$$

According to Formulas (4), (5), and (7), the output frequency of the humidity-sensing unit is

10 $${f}_{H}={f}_{{P}_{0},{H}_{0}}^{{T}_{0}}+\varDelta {f}_{P,H}^{T}-\varDelta {f}_{P,{H}_{i}}^{T}+\varDelta {f}_{{P}_{0},{H}_{0}}^{T}={f}_{{P}_{0},{H}_{0}}^{{T}_{0}}+{S}_{H}\times \varDelta H+\varDelta {f}_{{P}_{0},{H}_{0}}^{T}$$

In an atmospheric environment, the humidity-sensing unit increases:

11 $$\varDelta H=\frac{|{f}_{H}-{f}_{{P}_{0},{H}_{0}}^{{T}_{0}}|}{{S}_{H}}$$

**Fig. 8:**
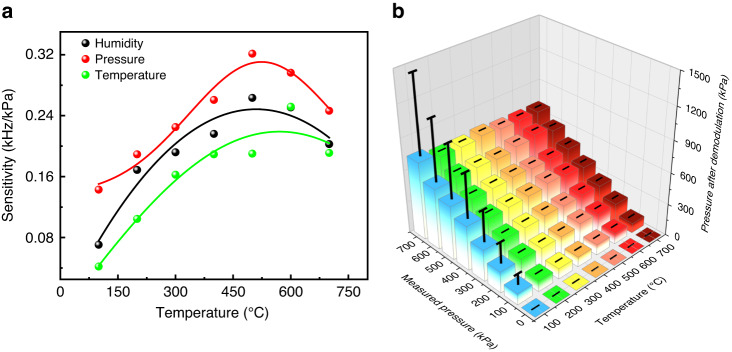
Error analysis of the integrated TPH sensor. **a** The relationship between the temperature and sensitivity of each sensing unit. **b** Error analysis between the measured value and the temperature demodulation value

The relationship between the sensitivity of each sensing unit and temperature is illustrated in Fig. 8a.

The relationship between temperature and sensitivity can be expressed with a Gaussian fit:

12 $$S={S}_{0}+A{e}^{-\frac{{(T-{T}_{c})}^{2}}{2{\omega }_{2}}}$$

As shown in Formula (12), as the temperature increases, the sensitivity increases first and then decreases, showing a quadratic form. The sensitivity of the pressure sensor varies greatly due to the simultaneous loading of temperature and pressure.

Error analysis between the measured value of the pressure-sensing unit and the demodulation value of the temperature is shown in Fig. 8b. The error increases with pressure, with a maximum error of 5.5%. Meanwhile, the compensation algorithm can effectively detect pressure values with reasonable accuracy and strong dependability.

## Conclusion

In summary, we report a temperature, pressure, and humidity coplanar sensor system by integrating LSG-based SAW sensors, which offer the advantages of miniaturization and multiparameter coplanar integration. In experiments, the MMG nanocomposite material was used as the humidity-sensitive layer to achieve high sensitivity and a fast response (3.18 s) and recovery time (0.94 s). Moreover, the design principle of the multiparameter coplanar-integrated SAW sensor was analyzed for preparing the TPH coplanar-integrated sensor. The experimental results indicate that the TPH SAW sensor can work reliably at 25–700 °C, 0–700 kPa, and 10-98% RH. When the temperature reaches 700 °C, the resonance frequency of the pressure-sensitive unit is offset by 172.5 kHz under the same humidity, and its sensitivity is 287.5 Hz/kPa. The sensitivity of the humidity-sensing unit is 3.77 KHz/%RH under low humidity (11%RH-83%RH) and 14.9 KHz/%RH under high humidity (83%RH-98%RH) conditions. In addition, a multiparameter decoupling algorithm is developed to address the coupling problem between multiple parameters in a complex environment to improve the measurement accuracy of the sensing units when multiple parameters are monitored. Therefore, the proposed TPH coplanar-integrated sensor has the potential to simultaneously measure multiple parameters in a harsh environment.

### Supplementary information

Supplementary data

## Data Availability

The data that support the findings of this study are available from the corresponding author upon reasonable request.
