# Supplementary material for: Temperature, pressure, and humidity SAW sensor based on coplanar integrated LGS
Source: Microsyst Nanoeng. 2023 Sep 11;9:110. doi: 10.1038/s41378-023-00586-0 (PMC10493225; doi:10.1038/s41378-023-00586-0)
Supplement: Supplementary file 1 — Supplementary data [file 41378_2023_586_MOESM1_ESM.docx]

**Supplementary Information**

**Temperature, Pressure, and Humidity SAW Sensor Based on Coplanar Integrated LGS**

Xiaorui Liang^1,2^, Lei Zhang^1,2^, Qiulin Tan^1,2,^*, Wenhua Cheng^1,2^, Dan Hu^1,2^, Shuang Li^1,2^, Lin Jing^3^, Jijun Xiong^1,2^

*^1^State Key Laboratory of Dynamic Measurement Technology, North University of China, Taiyuan 030051, China*

*^2^Key Laboratory of Micro/nano Devices and Systems, Ministry of Education, North University of China, Tai Yuan 030051, China*

*^3^School of Materials Science and Engineering, Nanyang Technological University, 50 Nanyang Avenue, Singapore 639798, Singapore*

******E-mail:* Qiulin Tan([tanqiulin@nuc.edu.cn](mailto:tanqiulin@nuc.edu.cn))

| Fig.S1 | Reasonable layout diagram of multi-parameter integrated sensor. | 3 |
| --- | --- | --- |
| Fig.S2 | **a** The image of comparison between the separated device and the connected devices. **b** The impedance curve of the device. | 4 |

**
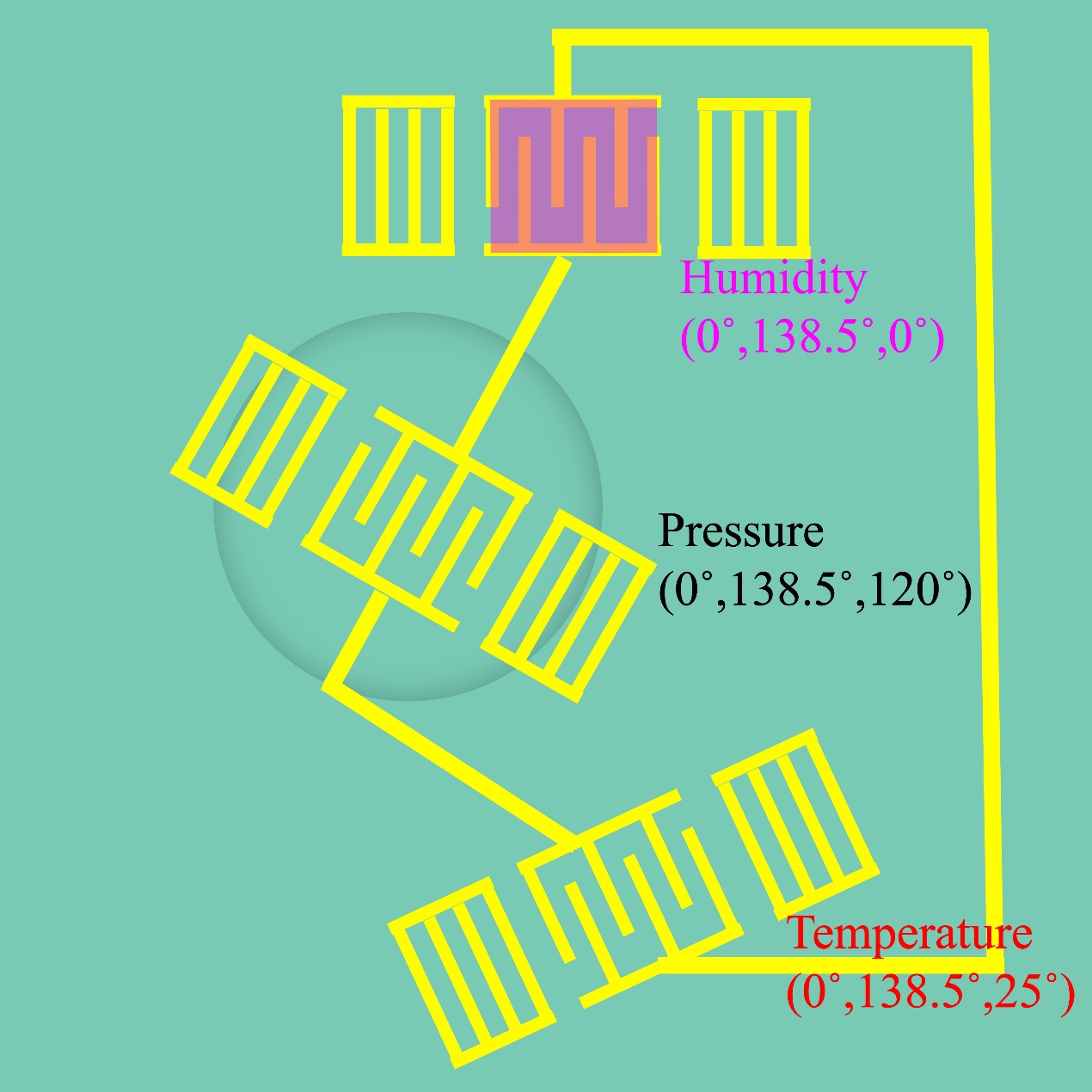
**

Fig.S1 Reasonable layout diagram of multi-parameter integrated sensor.

**
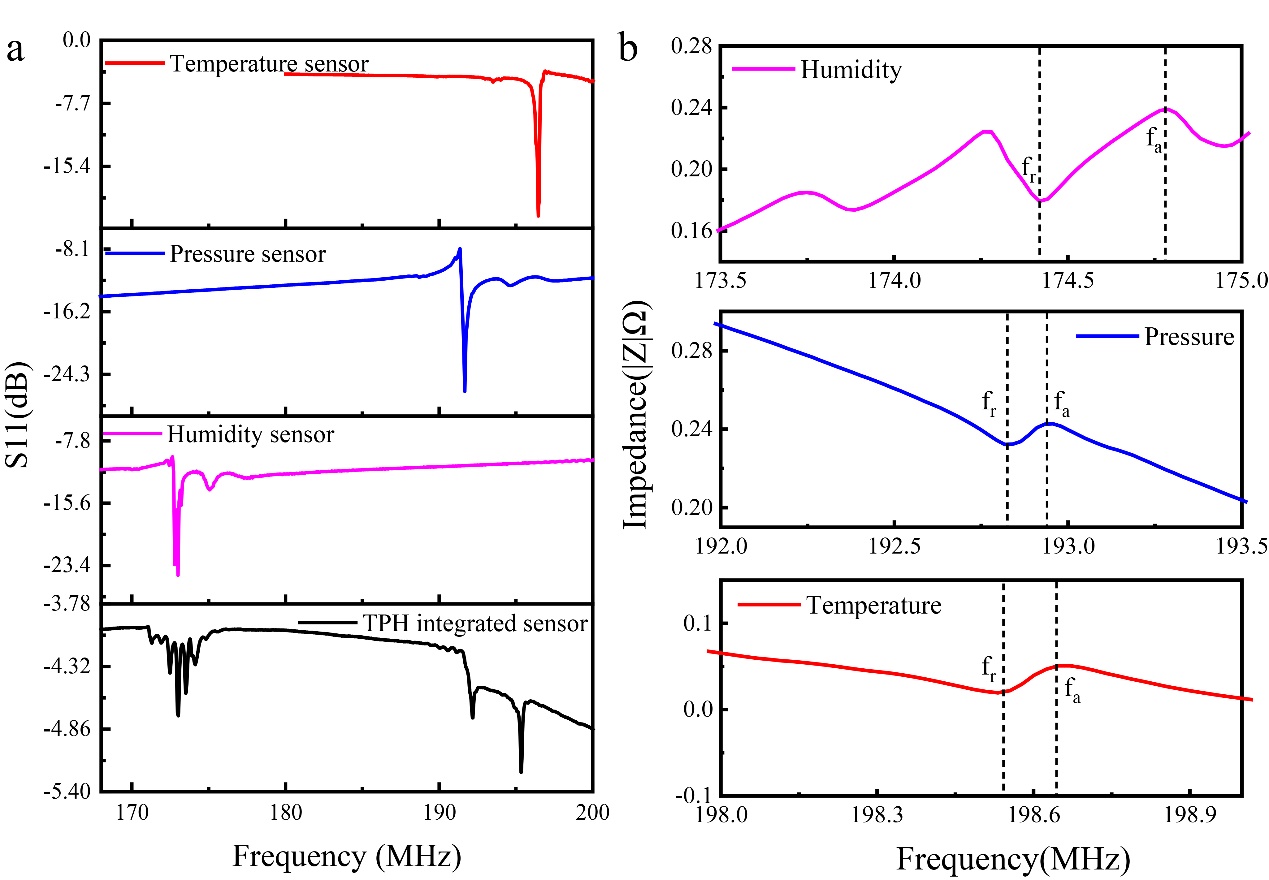
**

Fig.S2 **a** The image of comparison between the separated device and the connected devices. **b** The impedance curve of the device.
